# The Molecular Signature Related to Local Inflammatory and Immune Response in Canine Cutaneous Hypersensitivity Reactions: A Preliminary Study

**DOI:** 10.3390/cimb46080542

**Published:** 2024-08-22

**Authors:** Camilla Capaccia, Francesco Ciancabilla, Ilaria Porcellato, Chiara Brachelente, Massimo Zerani, Margherita Maranesi, Gabriella Guelfi

**Affiliations:** Department of Veterinary Medicine, University of Perugia, 06132 Perugia, Italy; camilla.capaccia@dottorandi.unipg.it (C.C.); francesco.ciancabilla@studenti.unipg.it (F.C.); chiara.brachelente@unipg.it (C.B.); massimo.zerani@unipg.it (M.Z.); gabriella.guelfi@unipg.it (G.G.)

**Keywords:** cutaneous hypersensitivity reactions, inflammatory–immune crosstalk, pattern recognition receptors, acute phase proteins, JAK/STAT pathway, canine skin

## Abstract

Cutaneous hypersensitivity reactions (CHRs) are complex inflammatory skin disorders that affect humans and dogs. This study examined the inflammatory and immune responses leading to skin damage, inflammation, and irritation by investigating gene expression through quantitative PCR (qPCR) and protein localization through the immunohistochemistry (IHC) of specific receptors and molecules involved in CHRs. Formalin-fixed paraffin-embedded (FFPE) samples from canine CHR skin (n = 20) and healthy dog skin (n = 3) were analyzed for expression levels of eight genes, including members of the pattern recognition receptor (PRR) family, CD209 and CLEC4G, the Regakine-1-like chemokine, and acute phase proteins (APPs), LBP-like and Hp-like genes. Additionally, we examined the local involvement of IL-6, Janus Kinase 1 (JAK1), and the signal transducer activator of transcription 3 (STAT3) in the CHR cases. The study demonstrated statistically significant increases in the expression levels of CD209, Hp-like (*p* < 0.01), LBP-like, Regakine-1-like, and CLEC4G (*p* < 0.05) genes in CHRs compared to healthy controls. Conversely, IL-6, JAK1, and STAT3 showed no significant difference between the two groups (*p* > 0.05). Protein analysis revealed JAK1 and STAT3 expression in CHR hyperplastic epithelial cells, dermal fibroblasts, and endothelial cells of small capillaries, indicating a possible involvement in the JAK/STAT pathway in local inflammatory response regulation. Our findings suggest that the skin plays a role in the development of CHRs.

## 1. Introduction

CHRs are common inflammatory skin disorders affecting humans and dogs [1,2]. The exact cause and pathogenesis of CHRs remains unknown. Diagnosis and treatment are complicated by the wide range of clinical manifestations, variable responses to therapy, and the overlap with other skin conditions [1,3]. CHRs are triggered by excessive immune reactions upon re-exposure to specific allergens, antigens, irritants, or infections, resulting in skin inflammation, damage, and irritation. CHRs encompass a variety of skin disorders including atopic dermatitis (AD), food allergies, parasitic hypersensitivity, contact hypersensitivity, and adverse drug reactions [1,2], with pruritus being the predominant symptom [4]. For diagnosis, canine CHR lesions are subjected to biopsy and subsequent histologic examination to differentiate the lesions from other conditions sharing similar clinical presentations and to assess tissue characteristics that either support or challenge the diagnosis [1].

The skin contains a sophisticated immune system comprising resident immune cells such as dendritic cells (DCs), macrophages, T cells, innate lymphoid cells, and skin cells like keratinocytes and melanocytes [5]. These cells form part of the skin-associated lymphoid tissue (SALT) [6], which defends against foreign microorganisms and environmental triggers by producing effector cytokines [7]. SALT also takes part in the pathophysiology of inflammatory states and hypersensitivity [8]. SALT reactions involve the activation of various immune cell types such as eosinophils and neutrophils, and the release of inflammatory mediators such as histamines, cytokines, and leukotrienes, which contribute to inflammation, skin damage, and irritation [4]. Genetic factors and environmental stimuli are believed to exacerbate an individual’s predisposition to these reactions [1,9].

The first response to restore CHR homeostasis is the acute phase response (APR), triggered by cutaneous intense immune and inflammatory processes [10,11]. Recent research has suggested that to regain skin health integrity and function, there exists a crosstalk between innate immune cells and inflammatory APPs [12,13]. The APR is a highly conserved system of inflammatory responses induced by PRRs present in skin cells (e.g., keratinocytes and resident immune cells) [14]. PRRs are pivotal in detecting specific molecular patterns associated with pathogens or tissue damage, and initiating responses to eliminate or contain the threat [15]. PRR engagement triggers the activation of intracellular signaling pathways, leading to a cascade of cytokine production that amplifies and regulates innate immunity and induces the production of APPs [15,16].

The APPs are critical in trapping microorganisms and their products, activating the complement system, sequestering cellular debris, neutralizing enzymes, scavenging free hemoglobin (Hb) and radicals [17], and regulating immune responses [18]. These proteins are synthesized mainly in the liver in response to proinflammatory cytokines, typically interleukin-1 (IL-1), interleukin-6 (IL-6), and tumor necrosis factor (TNF) [16]. Among the APPs, Haptoglobin (Hp) is a multifunctional protein whose concentration increases significantly during inflammation, infection, or injury [19,20]. Throughout the evolution of many mammals, a genetic duplication of a segment of the Hp gene resulted in a variation of the Hp gene known as zonulin. Zonulin, which originates from the HP2 allele and HP22 genotype, was identified in humans by Fasano et al. [21]. More recently, the zonulin variant has been associated with human AD [22]. This has been linked to zonulin’s role as a key regulator of skin and intestinal epithelial tight junction ultrastructure integrity [23].

Additionally, Lipopolysaccharide-binding protein (LBP), which binds lipopolysaccharide components of Gram-negative cell walls, garners attention as another significant APP, as its levels change locally and in the circulation in APR of inflammation [24].

A critical component in the regulation of immune responses in CHRs is the JAK/STAT signaling pathway, which includes the key genes JAK1 and STAT3. The JAK/STAT pathway is crucial for immune system functions, such as infection defense, immune tolerance, barrier strengthening, and cancer prevention. Many cytokines, including IL-6, involved in the pathogenesis of inflammatory diseases use this pathway to transduce intracellular signals. When cytokines bind to their receptors, they activate JAK, which then phosphorylates and activates STAT. Phosphorylated STAT (pSTAT) dimerizes and translocates into the nucleus to regulate the transcription of proinflammatory target genes. The JAK/STAT pathway is crucial for mediating responses to inflammatory signals and is involved in various cellular processes such as proliferation, differentiation, and apoptosis [25,26].

Growing evidence suggests that the dysregulation of the JAK/STAT pathway induces the expression of several critical mediators of inflammation [27] and cancer [28,29], and has a central role in the immunopathogenesis of inflammatory diseases including AD [25]. In AD, the JAK/STAT pathway regulates inflammation, epidermal barrier function, and peripheral nerve modulation associated with itch transduction [25]. Drug inhibition of the JAK/STAT pathway can attenuate AD symptoms and show clinical efficacy [30,31,32].

Considering these aspects, this study explores the prominent role of immune and inflammatory responses mediated by CD209 and CLEC4G, both members of the PRR family. The study also examines the Regakine-1-like chemokine, which, enhances the blood inflammatory response by synergizing with neutrophil chemoattractant, and then evaluates two APPs, LBP-like and Hp-like genes. In addition, the study investigates the gene and protein expression of IL-6 and the associated JAK1/STAT3 transcriptional mechanisms. The expression profile of the previously reported genes in healthy and CHR skin obtained from FFPE biopsies is assessed by qPCR. The JAK/STAT protein expression and localization in CHR and healthy FFPE cases are observed by IHC.

## 2. Materials and Methods

The research in this study was approved by the Ethical Committee of the University of Perugia, with protocol number: n.214499 dated 11 July 2022.

### 2.1. Study Objectives and Targeted Gene Selection

The first objective of the research was to investigate whether CHR was associated with the presence of the mature protein zonulin, an allelic variant of Hp2. Thus, in addition to qPCR evaluation of Hp gene expression, we genotyped Hp to reveal zonulin.

The second research objective was to study the local tissue response to CHR disease in dogs. Based on the knowledge of human CHR and the multifactorial mechanism regulating CHR, the crosstalk between APP and the immune response was investigated. After a detailed bioinformatic analysis of the pathways and based on the bibliography of the last five years, we focused our attention on the following factors:

- CD209 and CLEC4G, members of the PRR family;
- IL-6 and the Regakine-1-like chemokine, involved in innate immunity;
- the LBP-like and Hp-like genes, implicated in inflammatory APR;
- JAK1 and STAT3, involved in the signal transduction of diseases associated with immune and inflammatory responses.

### 2.2. Experimental Design

**Figure 1:**
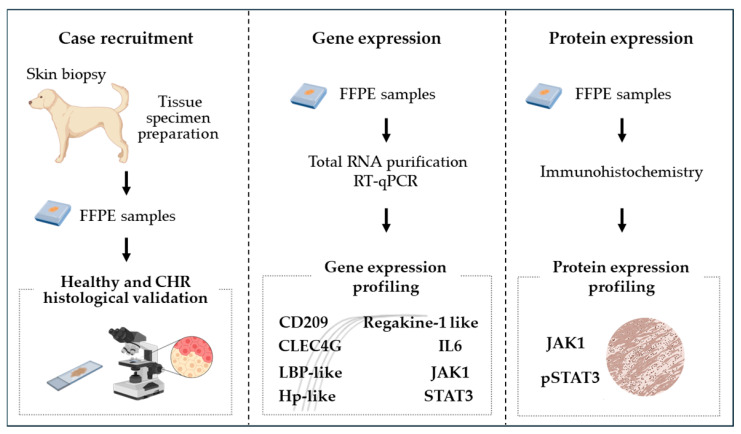
Experimental workflow. The study starts with canine skin biopsies and the preparation of FFPE specimens, which are then classified into healthy and CHRs based on histologic evaluation (case recruitment). Total RNA is extracted from the FFPE sample, and reverse transcribed, and target genes are amplified by qPCR (gene expression). JAK1 and pSTAT3 proteins are evaluated by immunohistochemistry (protein expression).

The study included a recruitment phase of FFPE samples from healthy and CHR cases, followed by qPCR evaluation of target gene expression profiles, and IHC protein evaluation (Figure 1).

### 2.3. Case Selection

The study was conducted on 23 canine FFPE specimens archived in the Pathology section of the Department of Veterinary Medicine (University of Perugia, Italy). Twenty cases of CHRs and three control cases were included in the study (Table 1). Samples had been collected as skin biopsies between 2019 and 2022. Two pathologists (I.P and C.B.) examined the samples, giving the histological diagnoses.

Inclusion criteria:

- Tissue samples on section > 0.5 cm^2^;
- Non-allergic dogs: no history, clinical signs, or microscopic findings suggestive of allergic dermatitis. Biopsy samples were collected from the slink of legs and face;
- Allergic dogs: clinical and histological findings compatible with hypersensitivity reactions. Biopsy samples were collected from the trunk (19/20 dogs) or the skin of the face and legs (1/20 dogs).

Exclusion Criteria:

- Dogs treated with corticosteroids, antihistamines, immunosuppressive drugs, or Janus kinase inhibitors within 14 days of the biopsy.

### 2.4. Total RNA Extraction, cDNA Synthesis, and qPCR Amplification

RNA was extracted from two FFPE sections (5 µm) using the FFPE-RNA Purification Kit (Norgen Biotek Corp, Thorold, ON, Canada) and stored at −80 °C until use [33]. RNA quality and quantity were assessed by spectrophotometry (NanoDrop™ 2000/2000 c, Thermo Fisher Scientific, Kandel, Germany) and fluorometry (Qubit RNA Assay, Life Technologies, Carlsbad, CA, USA). Total RNA (40 ng) was reverse transcribed in 20 μL of iSCRIPT cDNA (Bio-Rad, Hercules, CA, USA) according to manufacturer guidelines. To test genomic DNA contamination, controls without reverse transcriptase were included.

To increase the sensitivity of qPCR analysis, a pre-amplification step was performed using 3 μL of cDNA (diluted 1:10), 1 μL of TaqMan probes (Table 2), 10 μL of SsoAdvanced™ Preamp Supermix (Bio-Rad, Hercules, CA, USA), and water up to 20 μL. Preamplification reactions were run for 3 min at 95 °C, followed by 10 cycles of 15 s at 95 °C and 4 min at 58 °C. QPCR amplification was executed in a final volume of 20 μL using 10 μL of SsoAdvanced Universal Probes Supermix (Bio-Rad, Hercules, CA, USA), 1 μL of pre-amplification reaction, and 1 μL of TaqMan probes (Table 2).

QPCR cycling conditions were performed as described by Guelfi et al. [34]. Amplification was performed on the StepOne Plus Real-Time PCR System (Applied Biosystems, Foster City, CA, USA), and PCR amplification efficiency was calculated using StepOne Software v2.3 (Applied Biosystems). The Livak method was used to calculate the normalized value (2^−ΔCq^) of target genes [35]. All the Hp amplicons were sequenced to analyze genetic variations that justify zonulin presence [21,36,37].

### 2.5. Immunohistochemical Evaluation

From FFPE samples, 5 μm sections were cut and mounted on poly-l-lysine-coated slides, which then were dewaxed and dehydrated. Heat-induced epitope retrieval was performed in a microwave for 20 min in a Tris-EDTA buffer (pH 9.0). Immunohistochemical evaluation was performed on serial sections with antibodies reported to be cross-reactive in canine species, raised against phospho-STAT3 (pSTAT3, Tyr705) (Cell Signaling Technology, Danvers, MA, USA) and JAK1 (6G4) (Cell Signaling Technology Danvers, MA, USA). After incubation with primary antibodies (2 h), slides were treated with an ABC ready-to-use kit (Abcam, Cambridge, UK) according to the manufacturer’s instructions. The positive reaction was detected with 3-amino-9-ethylcarbazole (AEC), and Mayer’s hematoxylin was used as a counterstain. Positive controls were obtained from canine lymph nodes for both antibodies; negative controls were performed by omitting the primary antibody and incubating control sections with TBS [38,39]. The immunohistochemical protocols and antibodies used in this study are summarized in Table 3.

### 2.6. Statistical Analysis

Gene expression data were analyzed using a nonparametric Mann–Whitney test to compare the normalized expression values (2^−ΔCq^) of selected genes in healthy versus CHR samples. Statistical analyses were performed with GraphPad Prism 9 (GraphPad, San Diego, CA, USA). Statistical significance occurred when *p* < 0.05.

## 3. Results

### 3.1. Histological and Clinical Evaluation

Study details of the dogs are listed in Table 4.

### 3.2. Endogenous Reference Gene Selection and Normalized Gene Expression Profiling

The RNA 260/280 ratio was within 1.8–2.0. There was no significant difference in the yield of total RNA between samples. The normalized qPCR expression value was calculated using the 2^−ΔCt^ procedure, where ACTB is the reference endogenous control (ΔCt gene target = Ct target − Ct ACTB). Based on four mathematical approaches: comparative Delta-Ct, BestKeeper, NormFinder, and GeNorm, stability values were assigned to each endogenous control (EC). Then, the RefFinder algorithm ranked the most stable EC (ACTB) and the least stable EC (GAPDH and RPS18) based on the EC’s respective stability score (Table 5) [40].

**Figure 2:**
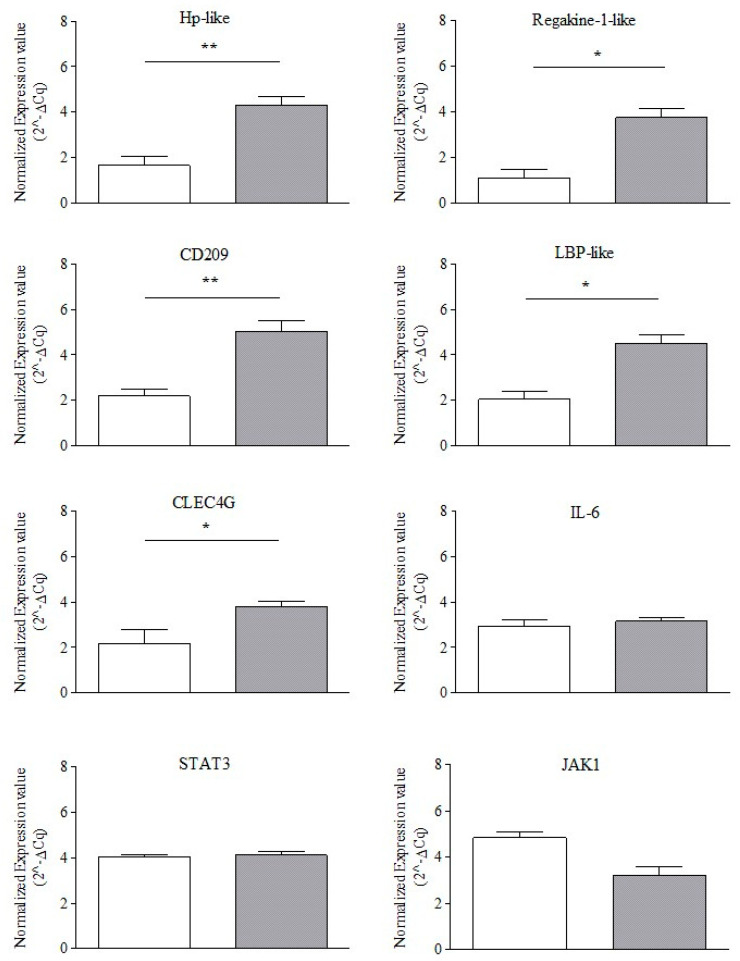
QPCR-normalized expression levels. The figure shows the normalized expression value (2^−ΔCq^) of target genes examined in the control (white column) and CHR (gray column) samples. The expression levels of Hp-like, Regakine-1-like, CD209, LBP-like, and CLEC4G genes exhibit statistically significant differences. Conversely, IL-6, JAK1, and STAT3 display no statistically significant difference between the two groups (*p* > 0.05). A non-parametric, unpaired, two-tailed Mann–Whitney test is employed for comparing the two groups of data (Control vs. CHR); * *p* < 0.05; ** *p* < 0.01.

All genes analyzed were present in the samples in a range of Cq values between 20–30 cycles. A comparison of the normalized gene expression between healthy controls (n = 3) and CHR cases (n = 20) revealed a statistically significant increase in the level of CD209 and Hp-like gene expression (*p* < 0.01) in CHR skin. Similarly, CLEC4G, Regakine-1-like, and LBP-like genes showed a statistically significant difference (*p* < 0.05). In contrast, IL-6, JAK1, and STAT3 showed no statistically significant difference between healthy and CHR cases (*p* > 0.05) (Figure 2).

All FFPE cases selected in the study were sequenced, and the sequences obtained were aligned with the Canis lupus familiaris haptoglobin-like mRNA sequence XM_038667180.1 deposited in the NCBI database. The sequence alignment revealed 100% identity, highlighting the absence of Hp gene variants which predict the involvement of an abnormal tight splicing protein (zonulin) responsible for intercellular adhesion failure.

### 3.3. pSTAT3 and JAK Protein Signalling and Localization

**Figure 3:**
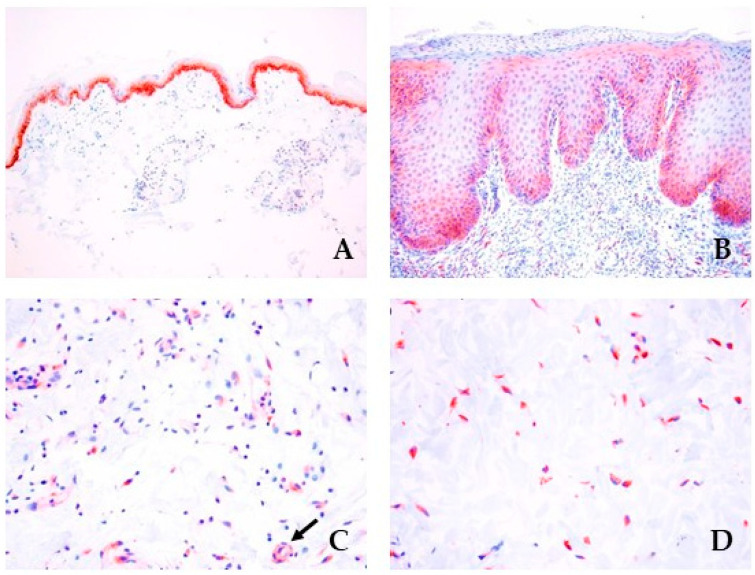
Immunohistochemical JAK1 expression. Expression of JAK1 within (**A**) healthy skin (AEC and hematoxylin, 10×) and (**B**) CHR skin (AEC and hematoxylin, 20×). Intense staining for JAK1 was observed in endothelial cells ((**C**), black arrow) and (**D**) fibroblasts in CHR samples (AEC and hematoxylin, 40×).

Expression of JAK1 was seen in the basal epithelium of normal controls (Figure 3A), whereas it showed diffuse immunolabeling in the hyperplastic epidermis of dogs with a CHR (Figure 3B). In CHR skin, a marked positivity was seen in the endothelial cells of capillaries (Figure 3C) and in the fibroblasts of the dermis (Figure 3D).

**Figure 4:**
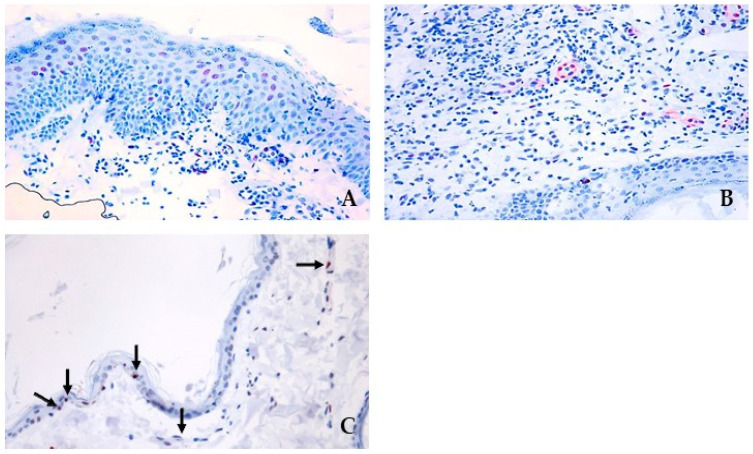
Immunohistochemical pSTAT3 expression. Expression of pSTAT3 within (**A**) hyperplastic epithelium in areas of moderate-to-severe inflammation (AEC and hematoxylin, 40×) and (**B**) endothelial cells in areas of inflammation (arrow, AEC and hematoxylin, 20×). On ((**C**), black arrows) healthy skin samples, pSTAT3 expression was seen only in rare cells within the epidermis and on vascular endothelial cells (AEC and hematoxylin, 40×).

A moderate nuclear positivity for pSTAT3 was present in the nuclei of the hyperplastic epithelia (Figure 4A) and the endothelial cells in small capillaries in the dermis, particularly within the areas of inflammation (Figure 4B).

## 4. Discussion

To the best of our knowledge, our study is the first to investigate the skin gene expression of CD209, CLEC4G, Regakine-1-like, Hp-like, LBP-like, IL-6, JAK1, and STAT3 genes, as well as the protein expression of JAK1 and STAT3 as a probable mechanism involved in canine CHRs. CHRs are inflammatory skin disorders commonly observed in veterinary practice concerning dogs. The immune system plays a central role in these disorders, where re-exposure to various triggers like allergens, irritants, or infections leads to a robust inflammatory APR. Components such as PRRs and APPs actively contribute to this immune response, exacerbating skin damage, inflammation, and irritation. Despite their common occurrence, understanding the underlying causes of CHRs remains elusive. Factors such as the integrity of the skin epithelial barrier, the skin microenvironment, and a complex interplay of genetic and environmental factors contribute to the diverse array of symptoms and individual susceptibility. This complexity poses challenges for accurate clinical diagnosis and impedes the development of effective treatments.

Recent studies on mammals, particularly humans, suggest that immune cells and inflammatory cytokines’ crosstalk in CHR conditions restore integrity, function, and skin health [5,12,13]. In primis, this crosstalk occurs locally at the lesion site. Locally in the SALT, when a pathogen breaches the cutaneous barriers, PRRs on sentinel macrophages and DCs recognize and bind the conserved molecular patterns in the pathogen, thanks to CD209, a C-type lectin receptor present on their surface [7]. Higher expression of CD209 initiates immune responses, activating the transcription of IL-6. In the skin, IL-6 is produced primarily by tissue-resident macrophages, keratinocytes, endothelial cells, and stromal cells [41,42]. The pleiotropic cytokine IL-6 upregulation, in turn, is the main response of the local APP upregulation [43]. Recently, many studies aimed at suppressing IL-6-induced APP gene expression have been conducted to evaluate the associated inflammatory responses [44].

The inflammatory state, when exacerbated, triggers the systemic hyperinflammatory state, often referred to as the “cytokine storm”, characterized by an increase in IL-6 blood levels [45]. The cytokine storm initiates a cascade of physiological changes leading to the loss of homeostasis and subsequent cellular damage. Additionally, the increase in systemic APP levels stimulates the pituitary–adrenal axis to release adrenocorticotropic hormone (ACTH) and the later cortisol in humans and corticosterone in rodents [46]. High cortisol levels contribute to reduced skin barrier function, impairing homeostasis and inflammation, and reducing the innate and adaptive immunity of the epidermis [47,48].

This study aimed to investigate the expression of CD209, CLEC4G, Regakine-1-like, Hp-like, LBP-like, IL-6, JAK1, and STAT3 genes in canine CHRs. The expression of CD209, CLEC4G, Regakine-1-like, Hp-like, and LBP-like genes in FFPE tissues was significantly increased in CHR cases compared to healthy subjects, highlighting their role in the disease. Our findings suggest that analyzing the expression of these genes in further studies could be clinically useful for diagnosing CHRs. This approach has the potential to improve upon traditional histological diagnosis, which relies on identifying a perivascular dermatitis pattern. However, this pattern is unfortunately nonspecific and can be present in many other skin diseases, limiting its diagnostic accuracy for CHRs. Most of the information on the potential role of CD209, CLEC4G, Regakine-1-like, Hp-like, and LBP-like genes and proteins comes from CHR studies in humans. One single transcriptomics study, although not validated by qPCR, exists in the canine literature, showing marked differences in the Canine Atopic Dermatitis (CAD) skin transcriptome compared with healthy controls. The study demonstrates, via Next Generation Sequencing (NGS), an upregulated fold change of CD209, CLEC4G, Regakine-1-like, Hp-like, and LBP-like genes in CAD. The authors attribute these results to the acute and chronic inflammatory process and immune component involvement but do not refer to the possible transduction mechanism [49].

The genes under investigation, CD209 and CLEC4G, possess a C-type carbohydrate recognition domain (CRD). Through this domain, they recognize specific carbohydrates present in pathogens, facilitating adhesion between cells and pathogens. In epithelial tissue, CD209 and CLEC4G are expressed on the surface of specialized dermal DCs. Several studies indicate that CD209, along with CLEC4G and TLR, activates the inflammatory response and regulates other functions of DCs to evoke immune responses [50,51].

Hp-like and LPB-like genes, which encode two proteins involved in the acute phase of inflammation, were found to be upregulated in CHR cases compared to controls, probably because they are induced by the proinflammatory cytokine IL-6 secreted by T lymphocytes and macrophages involved in the pathology [52]. For this reason, we aimed to investigate if, locally, there was a dysregulated expression of IL-6 as a probable modulator of the expression of CD209, CLEC4G, Regakine-1-like, Hp-like, and LBP-like genes via a JAK1/STAT3 signal transduction mechanism. IL-6 is a gene with a pleiotropic effect on inflammation, immune response, and hematopoiesis. Nevertheless, in our study, IL-6 did not reveal a statistically significant gene dysregulation. Our study focused on IL-6 gene expression, but IL-6 regulation is strictly controlled at the post-transcriptional level by several proteins in primis STAT3, and microRNAs. The activation of these proteins and microRNAs determines the fate of IL-6 mRNA [52]. In this regard, techniques like IHC might reveal a different picture of protein abundance. However, previous experience has led us to exclude the immunohistochemical assessment of the IL-6 protein simply because its pleiotropic role makes IL-6 highly tissue-present, making differential assessment impossible. Although IL-6 gene expression does not appear to be dysregulated in our study, previous human studies have shown that IL-6 induces Hp expression by activating STAT3 [53,,54]. The mechanisms regulating IL-6 in canine CHRs might differ from those observed in humans. Therefore, we cannot exclude that investigating IL-6 protein levels in CHR lesions using IHC could represent a future direction to understanding the specific role of IL-6 in canine CHRs. Also, the evaluation of the expression of IL-6 in other canine inflammatory skin diseases could elucidate whether the observed IL-6 expression pattern is unique to CHRs.

It is also known that an overexpression of Hp, which, like all APPs, is primarily produced in the liver, is functionally beneficial in inflammatory and immune diseases. Hp binds to Hb released from red blood cells during hemolysis, thereby preventing its harmful effects [55,56]. In CHRs, endothelial damage leads to the release of Hb, leading to an excess of free oxyHb, which contributes to aggravating endothelial damage by enhancing the inflammatory response [57,58]. Hp helps prevent Hb toxicity by structurally stabilizing the Hb molecule, which prevents the inflammatory and cytotoxic responses of the endothelial tissue. The Hb-Hp complex is then degraded by the CD163 scavenger receptor present in macrophages [59]. In the present study, we hypothesized that the overexpression of Hp mRNA could be justified by the need to counteract the toxicity of Hb due to CHR-associated inflammation.

In addition to studying the expression of the Hp gene, we investigated the association between CHRs and the possible presence of an Hp variant. To date, the canine homolog of the zonulin has not been identified, and therefore, the physiological role of canine zonulin in the CHR pathology remains unclear. In our study, the Hp gene was sequenced in all CHR cases to assess the presence of the Hp gene variant; the sequences exhibited 100% homology with the XM_845903 NCBI Reference Sequence, thus excluding this hypothesis.

Assuming that the upregulated CD209, CLEC4G, Regakine-1-like, Hp-like, and LBP-like genes in CHR pathology could be linked to the JAK/STAT transduction mechanism, we examined the gene expression and protein localization of JAK1 and STAT3 in the CHR skin of canine patients [60]. In recent years, the JAK/STAT pathway has also been extensively studied in various human skin diseases, such as AD [61], psoriasis [62], cutaneous lupus erythematosus [63], and pyoderma gangrenosum [61]. In addition, recent insights into this pathway have identified new therapeutic targets for inflammatory skin diseases [64]. In response to acute inflammation, JAK/STAT signaling plays a critical role in initiating and regulating innate and adaptive immune responses. However, if this mechanism is deregulated, it becomes maladaptive and leads to chronic inflammation, exacerbating the condition [65,66]. This cytokine-regulated mechanism, which itself upregulates cytokines, could explain the observed local pro-inflammatory CD209, CLEC4G, Regakine-1-like, Hp-like, and LBP-like gene upregulation.

We are not surprised that JAK1 and STAT3, in our study, do not show increased gene expression levels like the other genes observed in the study. The reason for this is that JAK and STAT are considered constitutive genes, and their positive regulation mainly occurs at the protein level through conformational changes and phosphorylation [67].

In light of this, we believed that it was interesting to examine the IHC protein expression and tissue localization of JAK1 and STAT3 in CHR dog skin. The study results showed JAK1 protein localization in the basal layer of the epidermis in healthy skin and diffuse JAK1 expression in the hyperplastic epidermis of CHR cases. The study also evidenced JAK1-positive cells in the dermal inflammatory infiltrate of the severely inflamed skin. Our findings partly align with those of Sartori et al., who reported a similar expression pattern in skin biopsies from AD beagles [68]. In terms of localization, JAK1 exhibited the expected cytoplasmic staining, consistent with the typical localization of JAK enzymes.

Similarly, pSTAT3 expression was observed in the nuclei of the hyperplastic epithelium within areas showing moderate-to-severe CHR inflammation. Immunohistochemical analysis confirmed the nuclear localization of pSTAT3, indicating that, after activation and phosphorylation, pSTAT3 translocates to the nucleus to enhance gene transcription or induction. This study demonstrates a correlation between JAK1 and STAT3 immunolocalization in the hyperplastic epidermis of CHR skin. The result suggests that they contribute to local epidermal hyperproliferation through the JAK/STAT pathway, as is known in other human skin diseases [69].

JAK1 and pSTAT3 also exhibited strong positivity in the endothelial cells of small dermal capillaries in areas with CHR inflammation. Vascular remodeling, a characteristic of inflammatory diseases such as chronic airway inflammation [70], rheumatoid arthritis [71], inflammatory bowel disease [72], and psoriasis [73], involves changes in vessel phenotype, resulting in a hyperpermeable, an enlarged vessel network with increased blood flow and infiltration of inflammatory cells. The JAK/STAT pathway contributes to endothelial dysfunction and prolonged vascular inflammation [74]. It is demonstrated that the APP/IL-6/JAK1/STAT3 signaling transduction pathway in human vascular endothelial cells induces capillary reshaping and releases pro-inflammatory chemokines attracting and enhancing the migration and infiltration of inflammatory cells and other cytokines to the site of inflammation [75]. Likely, in CHR skin, the activation of the JAK1/STAT3 pathway in endothelial cells promotes capillary remodeling and the infiltration of inflammatory mediators and immune cells to the inflamed skin.

**Figure 5:**
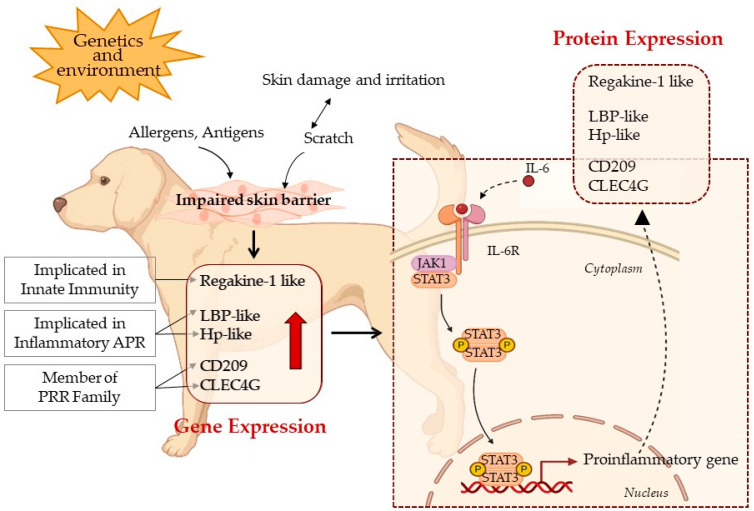
A hypothetical model of local immune and inflammatory crosstalk in canine CHRs. The CHR, locally, induces immune and inflammatory responses. The APR occurs as an acute non-specific defensive response in the presence of tissue damage of a various nature, such as allergens, and antigens. Itching, induced by the APR, causes the tissue damage of the epithelial barrier, amplifying the APR (figure at top left). At the tissue level, this condition results in the overexpression of Regakine-1-like, LBP-like, Hp-like, CD209, and CLEC4G genes. In the box with a continuous line (lower left), the up arrow indicates gene upregulation. The mechanism by which IL-6 binding to its receptor promotes the activation of JAK1 and STAT3 proteins is reported in the box with a dotted line at the lower right of the figure. The dimerized and phosphorylated STAT3 translocates to the nucleus, leading to the expression of proinflammatory genes. This triggers an amplification circuit of the inflammatory response by overexpressing Regakine-1-like, LBP-like, Hp-like, CD209, and CLEC4G genes. IL-6/JAK1/STAT3 also activates the production of pro-inflammatory cytokines, including IL-6. Cytokines, in turn, stimulate the inflammatory response, although our results suggest that IL-6 is not overexpressed as an mRNA. Considering that a CHR has a high epigenetic component, we hypothesize that there might be an activation of IL-6 protein expression due to an epigenetic mechanism related to the animal’s living conditions. The CHR cytokine storm can cause severe systemic effects by turning acute inflammation into chronic inflammation. The boxes with a solid line are derived from results obtained in qPCR experiments, while the boxes with a dashed line integrate IHC protein results with known processes in diseases of different mammals.

The immunohistochemical analysis of JAK1/pSTAT3 in CHR cases suggests that the increased expression of the CD209, CLEC4G, Regakine-1-like, Hp-like, and LBP-like genes is associated with a shift from a quiescent state to a proliferative phenotype. These findings suggest that the JAK1/STAT3 pathway may play a role in the pathogenesis, as indicated by its intense and diffuse expression in CHR cases (Figure 5).

The expression of the JAK1/STAT3 pathway in the CHR skin holds promise as a potential target for topical treatments. In human medicine, topical JAK inhibitors, such as tofacitinib, ruxolitinib, and brepocitinib, have effectively treated AD [76]. In veterinary medicine, oclacitinib, a JAK inhibitor that has been FDA-approved since 2013, is used to manage canine allergic skin diseases [77]. It improves pain and itching, reduces the severity of CAD signs, and selectively inhibits JAK1 from interfering primarily with allergic cytokine signaling.

## 5. Conclusions

This study represents a significant advancement in understanding canine CHR immunopathogenesis. Our findings demonstrate the significant upregulation of CD209, Hp-like, LBP-like, Regakine-1-like, and CLEC4G genes in CHR cases compared to healthy controls, indicating a complex interplay between immunological and inflammatory signals in the CHR pathogenesis. Protein analysis revealed the expression of JAK1 and pSTAT3 in hyperplastic epithelial cells, dermal fibroblasts, and endothelial cells of small capillaries in CHR cases, indicating a possible involvement of the JAK/STAT pathway in regulating local inflammatory responses. In conclusion, studying the genes and proteins expressed in CHRs provides new possibilities for identifying diagnostic biomarkers and developing innovative therapeutic approaches, ultimately contributing to better clinical management and the overall well-being of dogs affected by CHRs.

## Figures and Tables

**Table 1:** Case recruitment. The table lists the cases selected in the study, the CHR cases (n = 20), and the healthy controls (n = 3). Age (years), sex, and breed are reported for each subject. Abbreviations: M, male; F, female; NA, data not available.

CASE	AGE	SEX	BREED
1-Control	6	F	Jack Russell
2-Control	3	M	Mixed breed
3-Control	5	M	Mixed breed
4-CHR	12	F	Jack Russell
5-CHR	7	F	Jack Russell
6-CHR	13	M	Cane Corso
7-CHR	10	M	Shar Pei
8-CHR	5	M	Mixed breed
9-CHR	7	M	Chow Chow
10-CHR	2	M	Pitbull
11-CHR	4	M	Mixed breed
12-CHR	1	NA	Jack Russell
13-CHR	10	F	Yorkshire terrier
14-CHR	12	F	Mixed breed
15-CHR	16	M	French bulldog
16-CHR	6	F	Dachshund
17-CHR	12	M	King Charles Cavalier Spaniel
18-CHR	9	M	German shepherd
19-CHR	1	M	Pitbull
20-CHR	6	F	Maremma sheepdog
21-CHR	1.5	F	Poodle
22-CHR	1.5	F	Mixed breed
23-CHR	5	F	Mixed breed

**Table 2:** TaqMan probes. The table shows the probes used for qPCR amplification. The first column lists the gene name, the corresponding acronym, and the target sequence, followed by the TaqMan probe ID (second column). In the third column, the exon-exon junction is indicated. The bp amplicon length is indicated in the last column. The probe for the LBP-like gene is predicted in *Oryctolagus cuniculus*, but it exhibits 100% cross-reactivity with *Canis lupus*.

Target Gene	TaqMan	Exon	Amplicon
Actin Beta ACTB-NM_001195845.2	Cf04931159_m1	1–2	52
Glyceraldehyde-3-Phosphate Dehydrogenase GAPDH-NM_001003142.2)	Cf04419463_gH	5–6	54
Ribosomal Protein S18 RPS1-NM_001048082.1	Cf02681523_g1	3–4	160
Cluster of Differentiation 209 CD209-NM_001130832.1	Cf02638221_g1	7–8	63
C-Type Lectin Domain Family 4 Member G CLEC4G-XM_005632943.3	Cf02647953_m1	4–5	115
Regakine-1-like XM_537721.5	Cf02644954_m1	1–2	91
Haptoglobin-like Hp-like-XM_845903.5	Cf02630391_m1	3–4	71
Lipopolysaccharide-binding protein-like LBP-lik-XM_017342134.1	Oc06724131_m1	7–8	76
Interleukin-6 IL-6-NM_001003301.1	Cf02624153_m1	4–5	66
Signal transducer and activator of transcription 3 STAT3-XM_005624457.3	Cf02727924_m1		147

**Table 3:** Detailed protocol of the antibodies used in the study. This table summarizes the commercially available antibodies included in the study, the antigen retrieval method, and the dilution. The phospho-STAT3 antibody detects endogenous levels of STAT3 only when phosphorylated at tyrosine 705. Abbreviations: mAb, monoclonal antibody.

Antigen	Clone	Antigen Retrieval	Dilution	Manufacturer
JAK1	Rabbit mAb	Tris-Edta buffer, pH 9.0	1:400	Cell Signaling Technology, Danvers, MA, USA
pSTAT3	Rabbit mAb	Tris-Edta buffer, pH 9.0	1:100	Cell Signaling Technology

**Table 4:** Histologic characteristics and clinical diagnosis. The table shows the final clinical diagnosis, group assignment, histologic lesions, inflammatory status, epidermal features, and eventual additional features of the study cases.

Clinical Diagnosis	Localization of Lesions	Inflammation Pattern and Cellularity	Epidermis	Additional Findings
1-Control	None	None	None	None
2-Control	None	None	None	None
3-Control	None	None	None	None
4-CHR	Back	Perivascular mast cells, fewer eosinophils, lymphocytes and plasma cells	Moderate, irregular hyperplasia and hyperkeratosis	Moderate dermal edema
5-CHR	Ventrum, Axillae	Perivascular mast cells, fewer lymphocytes and occasional eosinophils	Severe, irregular hyperplasia with spongiosis	Subepidermal edema and lymphocytic exocytosis
6- CHR	Back	Perivascular mast cells, eosinophils, plasma cells and lymphocytes	Severe, irregular hyperplasia and hyperkeratosis	Multifocal moderate dermal edema
7-CHR	Back	Perivascular mast cells, fewer eosinophils, lymphocytes	Moderate, irregular hyperplasia and hyperkeratosis	Diffuse dermal edema
8-CHR	Ventrum, thighs	Perivascular mast cells, lymphocytes and rare eosinophils and neutrophils	Moderate, irregular hyperplasia and hyperkeratosis	Subepidermal edema
9-CHR	Multifocal, predominantly back	Perivascular mast cells, lymphocytes, plasma cells rare eosinophils	Severe hyperkeratosis	Moderate dermal edema
10-CHR	Multifocal, predominantly ventrum and thighs	Perivascular mast cells, lymphocytes, plasma cells rare eosinophils	Moderate and irregular hyerplasia and hyperkeratosis	Subepidermic fibrosis
11-CHR	Back	Perivascular mast cells, eosinophils, lymphocytes	Moderate and irregular hyerplasia and hyperkeratosis	
12-CHR	Ventrum, thighs	Perivascular to intestitial mast cells, eosinophils. Rare lymphocytes and neutrophils	Mild, regular hyperplasia and hyperkeratosis	Moderate dermal edema, ulceration and crusts
13-CHR	Dorsal part of the neck and thorax	Perivascular mast cells, lymphocytes and rare eosinophils	Mild, regular hyperplasia and hyperkeratosis	Severe edema with microhemorrages
14-CHR	Ventrum, thighs	Perivascular to intestitial mast cells, eosinophils. Occasional lymphocytes and plasma cells.	Severe, irregular hyperplasia and hyperkeratosis	Severe dermal edema
15-CHR	Multifocal, predominantly ventral	Perivascular mast cells, lymphocytes, plasma cells rare eosinophils	Severe, irregular hyperplasia and hyperkeratosis	Epidermal spongiosis with lymphocytic exocytosis; mural folliculitis
16-CHR	Interdigital spaces, face, abdomen	Perivascular mast cells, lymphocytes, plasma cells	Moderate and irregular hyerplasia and basket-wave hyperkeratosis	Moderate edema and chronic Periadenitis
17-CHR	Back, limbs, nose	Perivascular to intestitial mast cells, eosinophils, lymphocytes and rare plasma cells	Mild, regular hyperplasia and hyperkeratosis	
18-CHR	Abdomen, thighs, hindlimbs	Perivascular to intestitial mast cells, eosinophils. Occasional lymphocytes, plasma cells and neutrophils	Mild, regular hyperplasia and hyperkeratosis	Lymphocytic exocytosis
19-CHR	Multifocal, predominantly ventral	Perivascular mast cells, lymphocytes, plasma cells and occasional eosinophils	Mild, regular hyperplasia and hyperkeratosis	Intraepidermal pustules
20-CHR	Back, thorax and abdomen	Perivascular mast cells, lymphocytes and rare eosinophils	Mild, regular hyperplasia and hyperkeratosis	Superficial edema
21-CHR	Abdomen	Perivascular mast cells and lymphocytes	Mild, irregular hyperplasia and hyperkeratosis	Mild superficial edema
22-CHR	Abdomen	Perivascular mast cells and lymphocytes	Mild, regular hyperplasia and hyperkeratosis	Mild superficial edema
23-CHR	Ventrum, thighs	Perivascular to intestitial mast cells, eosinophils and histiocytes	Severe, irregular hyperplasia and hyperkeratosis	Severe dermal edema

**Table 5:** Reference endogenous control stability. The resulting data obtained from the four algorithms, Delta Ct, BestKeeper, NormFinder, and GeNorm, are evaluated with RefFinder, which ranks from the most stable EC (first column) to the least stable EC (second and third columns). Results obtained by the RefFinder algorithm are highlighted on a gray background.

ALGORITHMS	EC RANK		
Delta Ct	ACTB	GAPDH	RPS18
BestKeeper	GAPDH	ACTB	RPS18
NormFinder	ACTB	GAPDH	RPS18
GeNorm	ACTB	GAPDH	RPS18
RefFinder comprehensive ranking	ACTB	GAPDH	RPS18

## Data Availability

Data are contained within the article.
